# CD5-Negative Primary Mantle Cell Lymphoma Presenting with a Bilateral Conjunctival Mass: A Potential Diagnostic Pitfall

**DOI:** 10.3390/curroncol30010062

**Published:** 2023-01-06

**Authors:** Magda Zanelli, Alberto Lugli, Andrea Palicelli, Francesca Sanguedolce, Maurizio Zizzo, Camilla Cresta, Samuele Biancafarina, Giovanni Martino, Barbara Crescenzi, Saverio Pancetti, Giuseppe Broggi, Rosario Caltabiano, Luca Cimino, Cristina Mecucci, Stefano Ascani

**Affiliations:** 1 Pathology Unit, Azienda USL-IRCCS di Reggio Emilia, 42123 Reggio Emilia, Italy; 2 Department of Pathological Anatomy, Modena University Hospital, 41125 Modena, Italy; 3 Pathology Unit, Policlinico Riuniti, University of Foggia, 71122 Foggia, Italy; 4 Surgical Oncology Unit, Azienda USL-IRCCS di Reggio Emilia, 42123 Reggio Emilia, Italy; 5 Pathology Unit, Azienda Ospedaliera Santa Maria di Terni, University of Perugia, 05100 Terni, Italy; 6 Haematology Unit, CREO, Azienda Ospedaliera di Perugia, University of Perugia, 06129 Perugia, Italy; 7 Pathology Unit, Humanitas University, Pieve Emanuele, 20072 Milan, Italy; 8 Pathology Unit, Humanitas Research Hospital-IRCCS, Rozzano, 20089 Milan, Italy; 9 Department of Medical and Surgical Sciences and Advanced Technologies “G.F. Ingrassia” Anatomic Pathology, University of Catania, 95123 Catania, Italy; 10 Ocular Immunology Unit, Azienda USL-IRCCS di Reggio Emilia, 42123 Reggio Emilia, Italy; 11 Department of Surgery, Medicine, Dentistry and Morphological Sciences, University of Modena and Reggio Emilia, 41124 Modena, Italy

**Keywords:** ocular adnexa, mantle cell lymphoma, CD5, cyclin D1, t(11;14)

## Abstract

Mantle cell lymphoma is a B-cell malignancy, which, in its classic form, usually involves lymph nodes and extranodal sites, and, among the extranodal sites, the gastrointestinal tract and the Waldeyer’s ring are most prevalent. MCL is rarely reported in the ocular adnexa, a site more frequently affected by extranodal marginal zone B-cell lymphoma of mucosa-associated lymphoid tissue, which is a form of low-grade malignancy. The diagnosis of MCL presenting in the ocular adnexa requires special attention as its rarity in this location combined with the not uncommon CD5 negativity of the disease when occurring in the ocular adnexa, may lead the pathologist to overlook the diagnosis and misinterpret MCL as marginal zone B cell lymphoma, which has a totally different behavior. Herein, we present a case of primary bilateral conjunctival CD5-negative MCL in a patient having no other sites affected by lymphoma and we discuss possible diagnostic pitfalls.

## 1. Introduction

Ocular adnexal lymphomas (OALs) represent approximately 1–2% of all lymphomas and 8% of all extranodal non-Hodgkin lymphomas (NHLs) [1]. The ocular adnexa may be either the primary site of the lymphoma occurrence or may be secondarily involved by lymphomas arising at other sites [2,3]. Approximately 5% of patients affected by NHLs are estimated to develop ocular adnexal involvement during the course of the disease [3].

The majority (80–90%) of primary OALs are represented by extranodal marginal zone B-cell lymphomas (EMZLs) of mucosa-associated lymphoid tissue (MALT), a form of low-grade malignancy which, in the ocular adnexa, has been found to be often associated with Chlamydia psittaci infection [4,5].

Mantle cell lymphoma (MCL) represents a rare B-cell malignancy accounting for 3–10% of NHLs, characterized in over 95% of cases by the presence of the translocation t(11,14)(q13;q32) leading to the overexpression of cyclin D1 [6].

MCL usually shows aggressive behavior with poor response to treatment. However, it is currently recognized to be a rather heterogeneous disease. In addition to the classical nodal MCL, which usually shows aggressive behavior, the current WHO classification recognizes the leukemic, non-nodal form, which behaves in a more indolent fashion [6,7].

Classical nodal MCL is often associated with involvement of multiple extranodal sites, often the gastrointestinal tract (GIT) and the Waldeyer ring. On the other hand, isolated extranodal disease at diagnosis represents a rare occurrence in MCL [8].

MCL, both primary or secondary, is rarely reported in the ocular adnexa [9,10,11,12,13,14,15,16,17]. Herein we present a case of primary bilateral conjunctival CD5-negative MCL in a patient with no other site involved by lymphoma at the time of diagnosis.

CD5 negativity is reported in MCL, mainly in the non-nodal form; however, cases of CD5-negative MCL have also been described in the ocular adnexa [11].

CD5-negative MCL may be easily overlooked and needs to be distinguished from other CD5-negative small B-cell lymphomas, including EMZL, lymphoplasmacytic lymphoma (LPL) and follicular lymphoma (FL).

## 2. Case History

**Figure 1:**
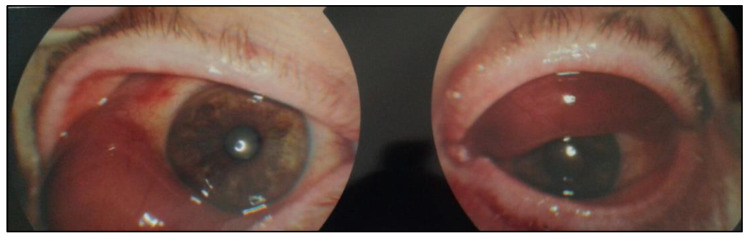
In vivo image showing bilateral conjunctival masses (original image from Dr E.P.).

A 78-year-old man, with a 4-year history of monoclonal gammopathy of undetermined significance (MGUS), presented with a palpable, painless, red, conjunctival lesion approximately 2 cm in diameter in his right eye (Figure 1).

His past medical history included cognitive impairment related to chronic vascular encephalopathy, post-myocardial infarction chronic heart failure, type 2 diabetes mellitus and dyslipidemia. Systemic symptoms, such as fever or night sweats, were absent. No palpable adenopathy was present. Blood tests were within normal limits with the exception of serum immunofixation showing a monoclonal IgG kappa component (14 mg/L). No anemia, hypercalcemia or signs of renal insufficiency were present.

Due to the increase of the right eye lesion and the onset of a similar lesion in the left eye, an ocular computed tomography (CT) scan without contrast was performed. Tissue of intermediate density, with lobulated outlines, surrounding the eyeballs and close to the eye muscles was detected. Based on these findings, the ophthalmologist suspected a lymphoproliferative disorder and a biopsy from the conjunctival lesion of the right eye was performed.

**Figure 2:**
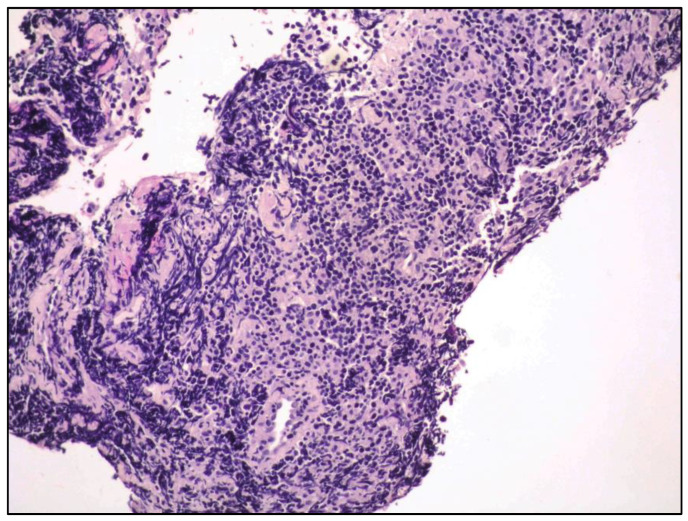
Medium power view showing a lymphoid proliferation composed of monotonous, small lymphocytes (hematoxylin and eosin; 100× magnification; original image from Prof. S.A.).

Histology revealed tiny fragments of conjunctival mucosa infiltrated by a lymphoid proliferation with a diffuse pattern of growth consisting of small lymphocytes (Figure 2).

**Figure 3:**
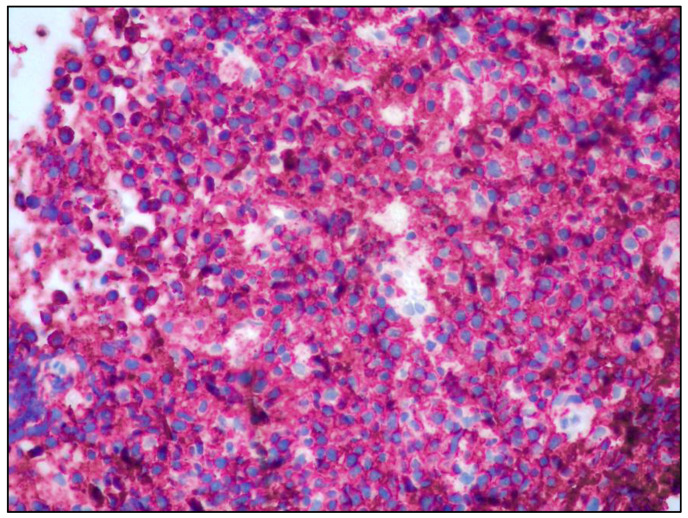
CD20 positivity highlighting the B-cell origin of the lymphoid proliferation (CD20 immunostaining; 200× magnification; original image from Prof. S.A.).

**Figure 4:**
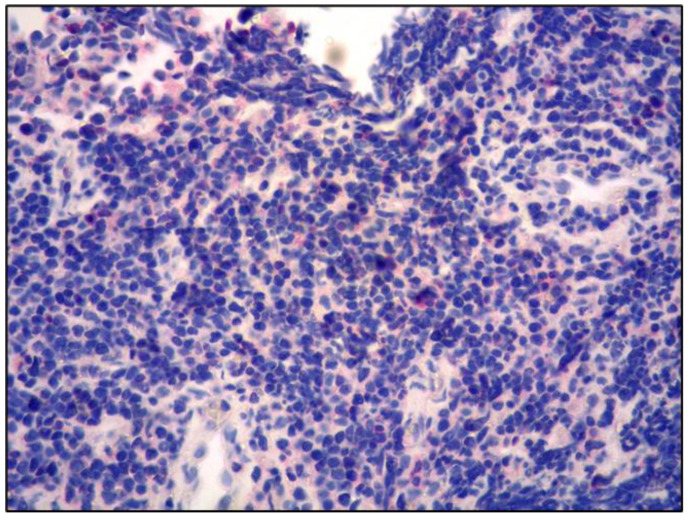
CD5 negativity of the lymphoid proliferation (CD5 immunostaining; 200× magnification; original image from Prof. S.A.).

**Figure 5:**
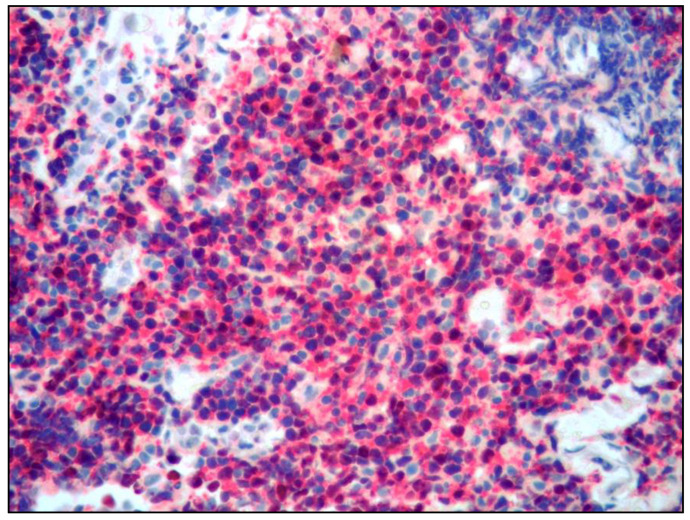
Diffuse cyclin D1 expression of the lymphoid proliferation (cyclin D1 immunostaining; 200× magnification; original image from Prof. S.A.).

The neoplastic lymphoid cells were positive for CD20, BCL2, SOX11 and cyclin D1 and negative for CD5, CD23, CD10, BCL6, IRTA1, CD138, MUM18 and CD3 (Figure 3, Figure 4 and Figure 5).

The proliferative index was low (Ki67/MIB1 about 5–10%).

By fluorescence in situ hybridization (FISH) analysis, a juxtaposition of IGH and CCND1 and a breakpoint in the CCND1 locus were detected in 76% of cells, indicating the presence of translocation t(11;14)(q13;q32). The histological and molecular analysis supported the diagnosis of MCL of classical type.

Clinical staging with total body CT scan and endoscopy of the gastrointestinal tract did not reveal any other site involved by lymphoma. BM biopsy confirmed the presence of MGUS, in absence of lymphoma. The patient started on chemo-immunotherapy with Bendamustine plus rituximab. Despite the rapid reduction in size of the ocular lesions, the patient developed bronchopneumonia and died shortly after the first cycle of treatment. During the course of the disease the control of the patient’s diabetes was not optimal and the immune suppression related to diabetes may have favored the occurrence of bronchopneumonia.

## 3. Discussion

Orbital lymphomas are subdivided into the following groups: primary OALs, which include lymphomas arising in the ocular adnexa, accounting for 1–2% of all NHLs [1]; secondary OALs comprising systemic lymphomas, involving the ocular adnexa at diagnosis or recurrence; and, finally, vitreo–retinal lymphomas, which are aggressive lymphomas, more often of B-cell origin and represent a sub-category of primary central nervous system lymphomas [18].

OAL is a relatively rare disease (approximately 8% of extranodal NHLs). However, lymphoma is one of the most common malignancies occurring in the ocular adnexa (55%) [1,17].

The majority of cases are lymphomas of B-cell origin and the most frequent subtype is EMZL (37–68%), followed by FL (10–23%), diffuse large B-cell lymphoma (DLBCL) (10–15%) and MCL (7–8%) [19,20,21]. The clinical features of OALs differ among the different subtypes, with MCL and DLBCL presenting with advanced disease stage and having a poor outcome, compared to EMZL and FL [17].

The majority of the data regarding OALs focuses on the most common histological subtype, that is EMZL. When clinicians and pathologists encounter less common histological subtypes, issues in their correct recognition and consequent management may arise.

MCL is a mature B-cell lymphoma deriving from the mantle zone of lymphoid follicles [6,7]. It comprises approximately 3% to 10% of NHLs arising in adult/elderly patients in western countries [6,7]. As already mentioned, the disease usually shows an aggressive, albeit heterogeneous, behavior with the classical form involving nodal and extranodal sites following a worse course, unlike the leukemic non-nodal counterpart, which has a more indolent behavior.

The diagnosis is established by the histological evaluation of the involved tissue, often characterized by a monomorphic proliferation of small- to medium-sized lymphocytes with slightly irregular nuclear contours and scant cytoplasm. MCL may show nodular, diffuse and more infrequently mantle-zone growth patterns and it may well show cytological variants, including blastoid, pleomorphic and marginal zone-like cells. The immunophenotype is rather characteristic with the expression of pan-B-cell markers (CD20, CD79alpha, PAX5), CD5, cyclin D1 and often SOX11, the latter of which is usually absent in the leukemic non-nodal form, which is biologically different from classic nodal MCL.

Cyclin D1 overexpression is a useful immunohistochemical marker for diagnosis as it represents a key event for MCL origin in the naïve pregerminal center B-cells and is associated with the translocation t(11;14)(q13;q32) leading to IGH:CCND1 fusion, identified approximately in 95% of cases [6,7]. Rare cyclin D1-negative MCL shows alternative cyclin activation by IG:CCDND2 fusion [19].

MCL involving ocular adnexa more often affects male, elderly individuals, and presents with bilateral (47%) orbital tumors. About 80% of MCLs presenting in the ocular region are found in advanced stages and the overall survival is reported to be around 57 months [17]. However, rare cases of MCL with an isolated extranodal disease, involving typical MALT sites (including the ocular adnexa), and following a more indolent course, have been recently reported by Morello et al. [8].

The majority of patients affected by ocular adnexa MCL show involvement of multiple structures of the ocular adnexa; however, orbital soft tissues are the most frequently involved structures (58%), followed by conjunctiva (31%), eyelid (16%), lacrimal gland (15%) and lacrimal sac (2%) [17]. A large study performed by Rasmussen et al. evaluated 230 cases of OALs from the Danish Ocular Lymphoma Database and identified 21 cases (9%) of MCL in the ocular area, with the most common site being the orbit, followed by the eyelid; 6 cases were localized in the conjunctiva [12].

Rasmussen et al. noted different behaviors between primary and secondary MCLs in the ocular area, as patients with involvement of the ocular adnexal region as the first presenting symptom of the disease presented more often with bilateral ocular tumors and BM involvement associated with inferior overall survival, compared to patients with secondary ocular MCL [12].

Primary conjunctival lymphomas account for 28% of OALs [1,2]. Jenkins et al. reported that conjunctival lymphomas have the lowest risk of dissemination compared to other orbital lymphomas [22].

Our patient presented with bilateral conjunctival masses in the absence of lymphadenopathy and a complete staging work-up did not reveal any other site involved with lymphoma, supporting the primary origin of the disease in the ocular area. Unlike MCL in the ocular adnexal region, usually characterized by disseminated disease at diagnosis, in our patient MCL was limited to the ocular region, showing similarities with the novel variant reported by Morello et al. [8].

It is intriguing that in our patient MCL affected both eyes, but no other site. In the case of EMZL, it is rather common tropism of this histological type of lymphoma for the ocular adnexa as it is now known that EMZL often involves sites where MALT is acquired. Therefore, we would not be surprised to have an EMZL involving the adnexa of both eyes in the absence of other sites. This event is much more uncommon for MCL. As previously mentioned, Morello et al. described a novel variant of MCL with isolated extranodal disease and a more indolent course than the classical nodal MCL [8]. This variant was defined by the authors as MALToma-like MCL. Comparing classical nodal MCL with MALToma-like MCL, it was noted that extranodal sites involved in classical nodal MCL were the GIT and the Waldeyer ring, while typical MALT sites were more often involved by MALT MCL [8].

From the histopathology point of view, MCL of the ocular adnexa recalls the morphological features of its nodal counterpart. However, in the ocular area some potential diagnostic pitfalls, leading the pathologists to overlook MCL, have to be considered

EMZL arising in the ocular adnexa may lack the lymphoepithelial lesions and the plasma cell component which are helpful diagnostic clues of EMZL arising in other sites, such as the stomach.

A morphological variant of MCL resembling marginal zone B-cell cell lymphoma and characterized by the presence of monocytoid B-cells with plasmacytoid features has been reported and may cause problems in differential diagnosis with EMZL [23]. In such cases, cyclin D1 overexpression is a reliable marker allowing the identification of MCL. Ponzoni et al. hypothesized that before cyclin D1 availability, cases of EMZL with unfavorable prognosis, could be unrecognized MCL [24].

Of note, in some cases immunohistochemistry may give confusing results.

CD5-positive EMZL may occur making the differential diagnosis with MCL difficult [25].

Additionally, about 5% of MCLs may lack CD5 expression [26]. Patients with CD5-negative MCL are often affected by the leukemic non-nodal form and are reported to have a more favorable outcome than CD5-positive MCL [26].

MCLs presenting in the ocular region are not uncommonly found to be CD5 negative, rendering their differential diagnosis from other CD5-negative small B-cell lymphomas, such as EMZL, which is much more common in the ocular adnexa [11,24], more difficult. In a study by Ferry et al., which analyzed 353 OALs, the authors identified 19 MCL cases, 3 of which were CD5 negative [27].

CD5-negative MCL needs to also be distinguished from LPL/Waldenstrom macroglobulinemia and FL. LPL is more polymorphic than MCL and usually shows a mixed population composed of lymphocytes, plasmacytoid lymphocytes and plasma cells. FL, composed of centrocytes and a variable proportion of centroblasts, shows the characteristic expression of follicle center markers, such as CD10 and BCL6. EMZL, LPL and FL are cyclin D1 negative and lack *CCND1* rearrangement.

In the present case, the negativity for CD5 required further analyses, including the assessment of cyclin D1 overexpression and the identification of t(11;14)(q13;q32) to confirm the diagnosis of MCL. Rare cases of ocular adnexal MCL have been reported to be cyclin D1 negative and, in such instances, SOX 11 positivity allows the recognition of MCL; the t(11;14) is also a useful diagnostic tool as it is detected in 95% of ocular adnexa MCLs [24].

The correct diagnosis of MCL is essential for the proper treatment which, in general, is based on immune-chemotherapy [11,27,28]. However, it has been suggested that rare and selected cases of MCL arising in MALT sites, with isolated extranodal disease, should be carefully evaluated to avoid possible overtreatment [8].

## 4. Conclusions

Despite representing only 1–2% of all NHLs, OAL is the most common orbital malignancy. EMZL, a low-grade B-cell lymphoma with an indolent course, is the most common histological subtype of lymphoma encountered in the ocular adnexa. MCL, often characterized by worse behavior and an advanced stage at presentation, rarely affects the ocular adnexa. The rare occurrence of MCL in the ocular area, combined with the often CD5 negativity of MCL arising at this site, may easily lead pathologists to overlook MCL of the ocular adnexa. In order to get to the correct diagnosis and adequate treatment, pathologists need to be aware of the potential diagnostic pitfalls.

## Data Availability

The data presented in this study is available in this article.
